# Feasibility of Using Intraoperative Neuromonitoring in the Prophylaxis of Dysesthesia in Transforaminal Endoscopic Discectomies of the Lumbar Spine

**DOI:** 10.3390/brainsci10080522

**Published:** 2020-08-05

**Authors:** Paulo Sérgio Teixeira de Carvalho, Max Rogério Freitas Ramos, Alcy Caio da Silva Meireles, Alexandre Peixoto, Paulo de Carvalho, Jorge Felipe Ramírez León, Anthony Yeung, Kai-Uwe Lewandrowski

**Affiliations:** 1 The Federal University of the State of Rio de Janeiro UNIRIO, Pain and Spine Minimally Invasive Surgery Service at Gaffrée Guinle University Hospital HUGG, Tijuca, Rio de Janeiro 20270-004 RJ, Brazil; neurocor8@gmail.com; 2 Federal University of the Rio de Janeiro State UNIRIO, Orthopedic Clinics at Gaffrée Guinle University Hospital HUGG, Tijuca, Rio de Janeiro 20270-004 RJ, Brazil; drmaxramos@hotmail.com; 3 The Meireles Neurology Institute, Bonsucesso - Rio de Janeiro 20230-240 RJ, Brazil; dralcycaio@hotmail.com; 4 Federal University of the Rio de Janeiro State UNIRIO, 775 – Maracanã, Rio de Janeiro 20270-004 RJ, Brazil; apeixotodemello@hotmail.com; 5 KRH Nordstadt Krankenhaus Hospital, 30167 Hanover, Germany; paulocarvalhoj@gmail.com; 6 Fundación Universitaria Sanitas, Clínica Reina Sofía-Clínica Colsanitas, Centro de Columna-Cirugía Mínima Invasiva, Bogotá 104-76, D.C., Colombia; jframirezl@yahoo.com; 7 Department of Neurosurgery Albuquerque, University of New Mexico School of Medicine, New Mexico Associate, Desert Institute for Spine Care, Phoenix, AZ 85020, USA; ayeung@sciatica.com; 8 Center for Advanced Spine Care of Southern Arizona and Surgical Institute of Tucson, AZ 85712, USA

**Keywords:** herniated disc, videoendoscopy, neuromonitoring, spine

## Abstract

(1) Background: Postoperative nerve root injury with dysesthesia is the most frequent sequela following lumbar endoscopic transforaminal discectomy. At times, it may be accompanied by transient and rarely by permanent motor weakness. The authors hypothesized that direct compression of the exiting nerve root and its dorsal root ganglion (DRG) by manipulating the working cannula or endoscopic instruments may play a role. (2) Objective: To assess whether intraoperative neurophysiological monitoring can help prevent nerve root injury by identifying neurophysiological events during the initial placement of the endoscopic working cannula and the directly visualized video endoscopic procedure. (3) Methods: The authors performed a retrospective chart review of 65 (35 female and 30 male) patients who underwent transforaminal endoscopic decompression for failed non-operative treatment of lumbar disc herniation from 2012 to 2020. The patients’ age ranged from 22 to 86 years, with an average of 51.75 years. Patients in the experimental group (32 patients) had intraoperative neurophysiological monitoring recordings using sensory evoked (SSEP), and transcranial motor evoked potentials (TCEP), those in the control group (32 patients) did not. The SSEP and TCMEP data were analyzed and correlated to the postoperative course, including dysesthesia and clinical outcomes using modified Macnab criteria, Oswestry disability index (ODI), visual analog scale (VAS) for leg and back pain. (4) Results: The surgical levels were L4/L5 in 44.6%, L5/S1 in 23.1%, and L3/L4 in 9.2%. Of the 65 patients, 56.9% (37/65) had surgery on the left, 36.9% (24/65) on the right, and the remaining 6.2% (4/65) underwent bilateral decompression. Postoperative dysesthesia occurred in 2 patients in the experimental and six patients in the control group. In the experimental neuromonitoring group, there was electrodiagnostic evidence of compression of the exiting nerve root’s DRG in 24 (72.7%) of the 32 patients after initial transforaminal placement of the working cannula. A 5% or more decrease and a 50% or more decrease in amplitude of SSEPs and TCEPs recordings of the exiting nerve root were resolved by repositioning the working cannula or by pausing the root manipulation until recovery to baseline, which typically occurred within an average of 1.15 min. In 15 of the 24 patients with such latency and amplitude changes, a foraminoplasty was performed before advancing the endoscopic working cannula via the transforaminal approach into the neuroforamen to avoid an impeding nerve root injury and postoperative dysesthesia. (5) Conclusion: Neuromonitoring enabled the intraoperative diagnosis of DRG compression during the initial transforaminal placement of the endoscopic working cannula. Future studies with more statistical power will have to investigate whether employing neuromonitoring to avoid intraoperative compression of the exiting nerve root is predictive of lower postoperative dysesthesia rates in patients undergoing videoendoscopic transforaminal discectomy.

## 1. Introduction

Videoendoscopy for the treatment of lumbar herniated discs is now common place with favorable clinical results comparable to microdiscectomy and a low rate of complication rate [1,2]. The transforaminal approach is frequently applied to the endoscopic treatment of herniated discs mainly at the L3/L4 and L4/L5 level. The approach is also feasible at L5/S1 but may be technically more demanding because of the configuration of the iliac wing, sacralization of the L5 vertebral body, or because of degenerative vertical collapse of the spine [3,4]. Placing the working cannula may be harder at this transitional level due to steeper attack angles making injury to the exiting L5 nerve root more likely. Regardless of the level, additional risks for nerve root injury due to increasing surgery time and more aggressive manipulation may arise if the surgeon is attempting to access a highly stenotic neuroforamen during the endoscopic decompression procedure. Compression of the dorsal root ganglion of the exiting nerve root may occur due to manipulation of the beveled endoscopic working cannula leading to temporary ischemia and postoperative dysesthesias in a percentage considerable number of patients [5].

Neuromonitoring could aid in mitigating these risk particularly if surgeons perform the lumbar transforaminal endoscopic surgery under general anesthesia [6]. Therefore, the authors decided to retrospectively study the result obtained by using neuromonitoring during transforaminal videoendoscopy in patients who were treated for herniated discs. While the three senior key opinion leader (KOL) surgeons (JFRL, ATY, and KUL) of this article have performed over 18,000 endoscopic spine surgeries between them [7,8,9,10,11,12,13,14,15,16,17,18] under monitored local anesthesia care (MAC) and sedation [12,18,19] with minimal complications [1,20] and utilizing the patients’ direct intraoperative feedback to treat validated pain generators [11,15,19,21,22], they recognize that neuromonitoring in some countries and in some clinical settings is the standard of care [23,24,25,26,27,28,29,30,31,32] in spite of added cost [33]. In some instances, it may even be a matter of necessity if MAC protocols for spine surgery are not supported by the local anesthesia teams. Therefore, the authors wanted to study the feasibility of identifying intraoperative neuromonitoring events that could be predictive of postoperative dysesthesias with the transforaminal endoscopic decompression procedure [34]. If feasible, surgical protocols aimed at lowering the incidence or perhaps even preventing injury to the exiting nerve root DRG could be developed for those surgeons who are unable to perform the surgery under local anesthesia with sedation [35].

## 2. Material and Method

### 2.1. Patients

**Figure 1:**
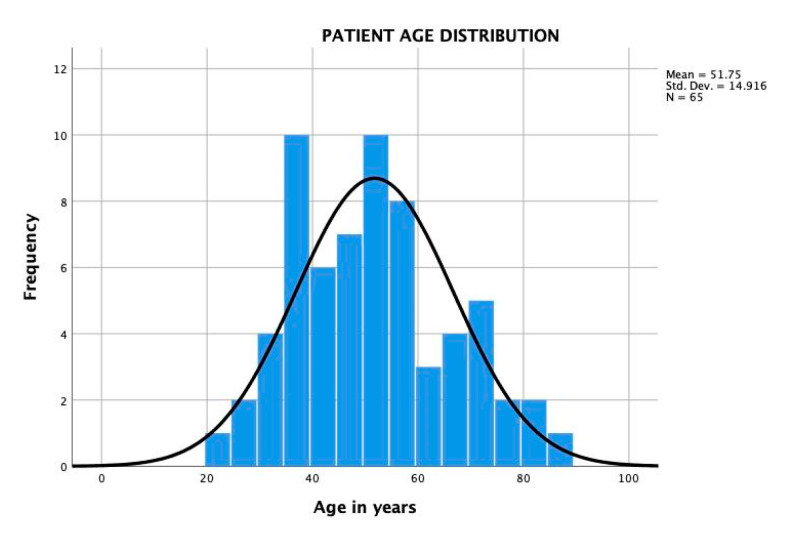
Age distribution of the 65 study patients with the superimposed expected normal distribution (black line). Patient’s age ranged from 22 to 86 years of age and averaged 51.75 years with a standard deviation of 14.916.

**Figure 2:**
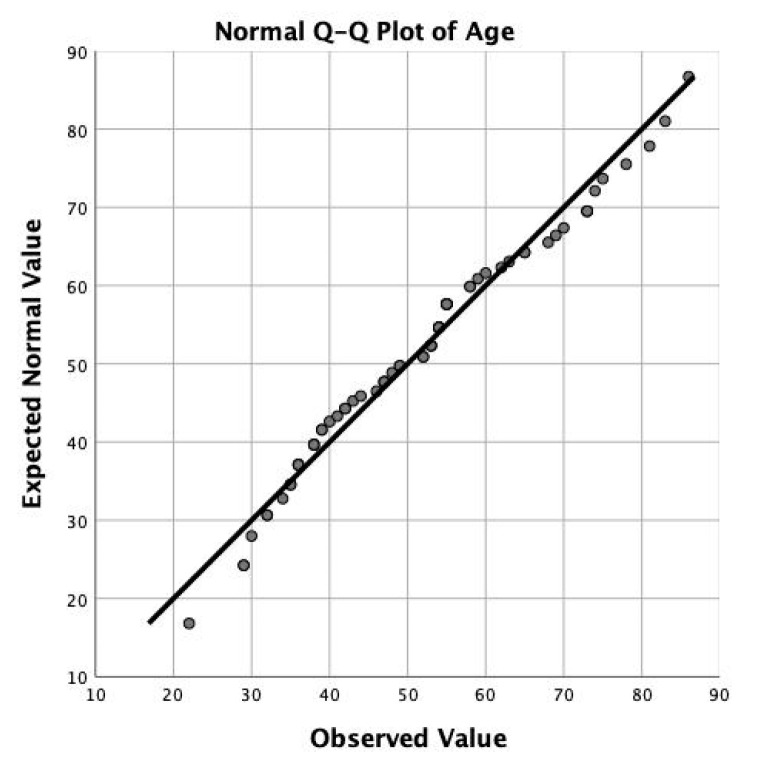
The quantile-quantile plot of the endoscopy study patients’ age shows normal distribution. The average age was 51.75 ± 14.916 years ranging from 22 to 86 years.

The charts, imaging studies, and neuromonitoring examinations of 65 patients who underwent transforaminal endoscopic discectomy from 2012 to 2020 were retrospectively reviewed. The surgeries were performed by the first and second author. There were 35 female (53.8%) and 30 (46.2%) male patients. There were 35 (53.8%) females and another 30 (46.2%) male patients. The average age was 51.75 years ranging from 22 to 86 years with a standard deviation (SDV) of 14.916 and followed normal distribution (Figure 1 and Figure 2). Patients in the experimental group (32 patients) had intraoperative neurophysiological monitoring recordings using sensory evoked (SSEP), and transcranial motor evoked potentials (TCEP), those in the control group (32 patients) did not. Both groups were age and gender matched, and similar rates of comorbidities, and surgical level distribution (see below). All patients in this consecutive case series provided informed consent and Institutional Review Board approval was obtained (CEIFUS 106-19). Written informed consent was obtained from the patient for publication of this report and any accompanying images.

### 2.2. Inclusion/Exclusion Criteria

Only patients presenting with radicular pain with a herniated disc confirmed by magnetic resonance imaging (MRI) or computed tomography (CT) were selected for this study. Patients with unmanageable radicular pain unresponsive to a minimum of 12 weeks of medical and interventional conservative care, with a positive Lasègue’s tension sign, and minimal low back pain were included. A normal preoperative electroneuromyographic study was another prerequisite for study inclusion. Patients with instability, deformity, or any electrodiagnostic abnormality were excluded from the study. Exclusion of patients from the study was prompted by a concurrent diagnosis of infection, tumor, or metastatic disease, or any electrodiagnostic evidence of chronic demyelination, or deinnervation in the dermatomes innervated by the affected surgical nerve roots. Exclusion was also prompted if patients displayed any of the following radiographic parameters.

### 2.3. Preoperative Radiographic Evaluation

Radiographic stenosis parameters were also evaluated. These included the posterior intervertebral disc and foraminal height [36]. Crossectional imaging showing 15 mm or less for the height of the neuroforamen, 3 mm or less measured as posterior intervertebral disc height, or the width of the neuroforamen was recorded as abnormal [36]. As previously published and validated, diagnostic selective nerve root blocks were used to determine the symptomatic painful level. This protocol was highly relevant in choosing the surgical level(s) in patients with multilevel disease [37,38,39,40,41,42,43,44]. Exclusion of patients from the study was prompted by a concurrent diagnosis of overt spondylolisthesis with more than 3 mm of translational motion on dynamic extension/flexion views. If patients were suspected of having claudication or mechanical back pain symptoms due to severe central stenosis, <100 mm^2^ at the surgical level or from facet arthropathy, they were typically also excluded from the study.

### 2.4. Anesthesia and Neuromonitoring

**Figure 3:**
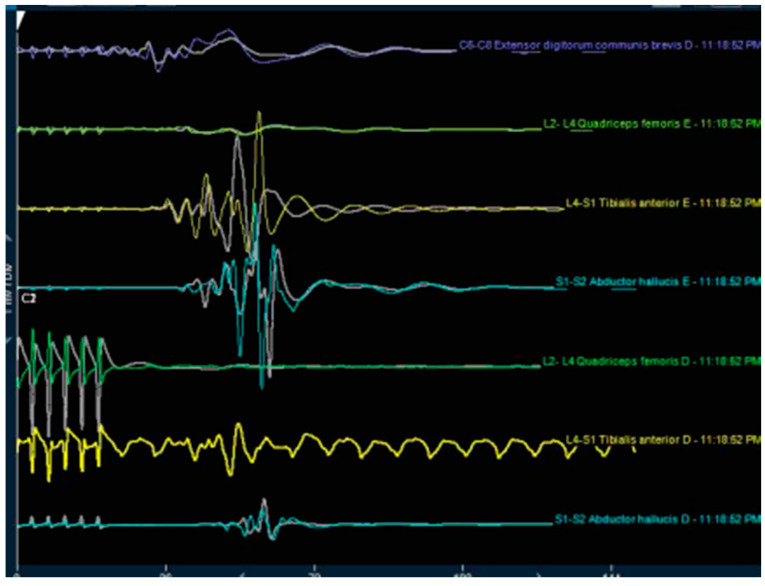
Preoperative examination (waves decreased in right anterior tibial and abductor of halux).

**Figure 4:**
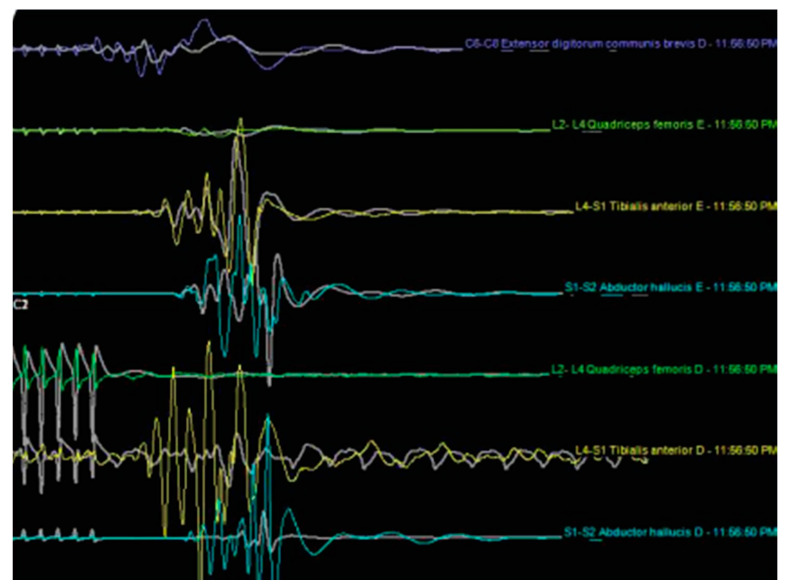
Postoperative examination (waves with amplitudes recovered in right anterior tibial and abductor of halux).

All patients underwent general anesthesia before the placement of the neuromonitoring probes in the lower limbs. Leads were placed in dermatomes corresponding to the roots exiting from the surgical level. The dermatome innervated by the level above the surgical segment was also monitored as a control. The neurophysiological monitoring was performed by recording using sensory evoked (SSEP), and transcranial motor evoked potentials (TCEP). Intra- and postoperative wave recordings were analyzed for changes in the frequency, amplitude, and latency (Figure 3 and Figure 4). A 5% increase or more in the SSEP latency or a 50% reduction in the amplitude was considered intraoperative evidence of a significant neuromonitoring event, suggesting injury to the DRG of the exiting nerve root. For the TCMEP, these parameters were a similar increase in latency, a decrease in the number of phases, and a 50% decrease in the amplitude. Conversely, a 50% increase in the SSEP and TCMEP amplitude, or a 10% latency decrease, was considered a significant improvement of the neuromonitoring parameters. The SSEP and TCMEP voltage improvements were recorded. Adverse neuromonitoring exiting nerve root compression events were analyzed and correlated to postoperative course including dysesthesia and outcome. In the case of a neuromonitoring event presumably due to compression or ischemia of the exiting nerve root, the surgery described below was paused, and the working cannula was withdrawn and then repositioned. The operation was resumed when neuromonitoring response caused by the surgical manipulation had returned at minimum to baseline. Foraminoplasty was performed in those patients where SSEP and TCMEP recordings did not return to baseline or were observed repeatedly with attempts to reposition the working cannula.

### 2.5. Endoscopic Discectomy Procedure

The surgeries were performed by the first and second author employing the transforaminal “outside-in” technique with the patient in prone position [45]. Serial dilation was employed to place the working cannula. Since patients with bony foraminal stenosis were excluded from the study, a routine foraminoplasty was not necessary. If bleeding occurred, a radiofrequency probe (Elliquence^®^ Baldwin, NY) was used for coagulation [46]. The endoscopic decompression procedure was directly visualized throughout the surgery. The location of the herniated disc and the presence of any other anatomical anomalies leading to inflammation or tethering the nerve roots bordering the triangular safe zone at the surgical level were recorded as the authors thought that they potentially could increase the risk of nerve root injury and postoperative development of irritation of the dorsal root ganglion (DRG). Fluoroscopic surveillance images were occasionally taken for orientation and verification of the decompression.

### 2.6. Clinical Follow-Up and Primary Outcome Measures

Primary clinical outcome measures were reductions in the visual-analog scales (VAS) [47] for leg (VAS-LEG) and back pain (VAS-BACK) ranging from no pain (0) to worst pain (10) and the Oswestry disability index ODI. The ODI is a ten-item composite instrument assessing pain intensity, personal care, and function including walking, lifting, personal care, sitting, standing, sleeping, social interaction, and traveling [48,49]. Each ODI item is scored from 0 (no impairment) to 5 (worst impairment). The individual scores are summed and then multiplied by two to obtain the ODI index ranging from 0 to 100. In addition, patients were evaluated with use of the modified Macnab criteria as [50,51]. Postoperatively, patients were seen in follow-up for reevaluation at 6 weeks and then at 3, 12, and 24 months. Any clinical evidence of new onset of dysethetic leg pain due to DRG irritation was recorded.

### 2.7. Postoperative Rehabilitation

Most patients did not require postoperative rehabilitation and supportive care requirements. Study patients treated for any acute onset of dysesthetic leg pain after an initial postoperative period of good pain relief with nonsteroidal anti-inflammatories, gabapentin, and transforaminal epidural steroid injections (TESI) pain syndromes were counted as having an irritation of the dorsal root ganglion (DRG). For the purpose of this study analysis, successful postoperative administration of 1% lidocaine-containing TESI with therapeutic pain relief was considered proof of a DRG irritation due to intraoperative nerve root injury.

### 2.8. Statistical Analysis

For the clinical outcome analysis, descriptive statistics (mean and standard deviation), cross-tabulation statistics of sensitivity, specificity, and measures of association were computed for two-way tables using IBM SPSS Statistics software, Chicago, IL, Version 26.0. The Pearson χ^2^ and the likelihood-ratio χ^2^ tests were used as statistical measures of association between dysesthesia, SSEP, TCMEP neuromonitoring events, and clinical outcome measures. The confidence intervals for the likelihood ratios were calculated using the “log method” according to Liberati and Altman et al. [52].

## 3. Results

Sixty-five study patients in total had surgery at 65 levels. There were 32 patients in the neuromonitoring and 33 patients in the control group. The average postoperative follow-up was 20.55 months, ranging from 12 to 30 months, with a SDV of 7.537 months. Most patients underwent surgery on the left side with 56.9% (37/65), whereas 36.9% (24/65) had surgery on the right, and the remaining 6.2% (4/65) of patients underwent bilateral surgery. As expected, the most common surgical segments were L4/L5 in 44.6%, L5/S1 in 23.1%, and L3/L4 in 9.2% of patients, respectively. The surgical level distribution is shown in Table 1. Eight (12.3%) of the 65 patients developed a postoperative dysesthesia from compression of the exiting nerve root and injury to its DRG during initial endoscopic working cannula placement. Crosstabulation of dysesthesia versus dichotomized age of less than 41 (*p* = 0.248) and greater than 41 years (*p* = 0.330) did not shown any statistically significant impact of age on the dysesthesia rate on Chi-square testing.

Final clinical outcomes were favorable, with 51 (78.5%) patients reporting excellent and the remaining 14 (21.5%) patients indicating good Macnab outcomes. At final follow-up, 75% (6 patients) of dysesthesia patient had excellent and the remaining 25% (2 patients) had good Macnab outcomes. At the final follow-up, the ODI improved from 31.71 ± 16.17 preoperatively to 19.02 ± 8.96 postoperatively (*p* < 0.0001). The VAS leg score reduced from 8.86 ± 0.93 before thee endoscopic decompression to 1.15 ± to 1.27 at the final follow-up (*p* < 0.0001). The VAS back score reductions were more modest from preoperative 4.92 ± 1.27 to postoperative 3.2 ± 0.775, respectively (*p* < 0.0001). The results of the paired-T testing with 95% confidence interval numbers are summarized in Table 2 and Table 3. The mean ODI reduction was 12.69 ± 13.12, on par with reported MCID (Minimal Clinically Important Difference) reductions reported for the transforaminal endoscopic decompression procedure. The mean VAS leg score reduction was 7.71 ± 1.9, being much larger than the reported MCID with the procedure. The mean VAS back reduction of 1.72 ± 1.21 did fall short of MCID numbers reported for this outcome tool with the transforaminal endoscopic procedure.

There were also statistically significant (*p* < 0.0001) improvements of the mean SSEP and TCMEP voltage measurements as a result of the transforaminal endoscopic decompression procedure (Figure 1). There were 24 (72.7%) of the 33 patients who underwent SSEP and TCMEP neuromonitoring during the transforaminal endoscopy, which showed a signal response due to compression of the DRG of the exiting nerve root during the initial position of the working cannula. In 14 (58.3%) of these 24 patients, withdrawing, waiting, and repositioning the endoscopic working cannula did not eliminate the intraoperative compression injury visibly demonstrated by the neuromonitoring. Hence, these patients underwent a foraminoplasty, which allowed the surgeon to advance the working cannula into the neuroforamen without visible irritation of the exiting nerve root’s DRG. The average waiting time until SSEP and TCMEP signals had normalized to prior baseline after irritation of the DRG of the exiting nerve root during the initial placement of the working cannular was 1.15 min. While the dysesthesia rate in the neuromonitoring (experimental) group was one-third of that observed in the control group, the authors’ study did not have sufficient statistical power to demonstrate the benefit with statistical significance (Pearson Chi-square = 0.557; *p* = 0.456).

## 4. Discussion

One of the most common problems after transforaminal endoscopic decompression is dysesthesia due to compression of the dorsal root ganglion by the working cannula and its manipulation during its initial placement and the discectomy or foraminoplasty procedure. The senior author reported the incidence of this unavoidable sequala at 12.45% based on a study in 1839 patients with foraminal stenosis being a statistically significant risk factor [1]. The last three authors of this article have performed the endoscopic transforaminal decompression under local anesthesia using MAC protocols to be able to use patient feedback during surgery while treating the predominant pain generator [11,22]. Their reported dysesthesia rate was lower [1,8,10] than reported by other surgeons in another multicenter study with seven participating surgeons and sites [53]. They reported the incidence of DRG irritation to average 21.5%, ranging from 5% to 41.2% in patients with excellent and good Macnab outcomes [53]. DRG irritation rates were independent of surgical level but highly dependent on surgeon skill level.

While the course of postoperative DRG irritation following a transforaminal endoscopic discectomy is benign and typically self-limiting with a reduced physical activity program, supportive medical and interventional care measures such as gabapentin, pregabalin, or a transforaminal epidural steroid injection (TESI), the condition can be quite annoying to patients until it resolves typically 2 to 4 weeks postoperatively [53]. A preoperative education program warning patients of the possibility of burning leg pain developing in the dermatome of the exiting nerve root of the surgical level some 5 to 10 days following the transforaminal endoscopic discectomy after an initial pain-free interval. Patients should also be instructed to avoid narcotic pain medication as they are not an effective treatment for this condition and that follow up with their surgeon in the office setting is more appropriate than seeking help in the emergency room where inconsequential imaging studies ultimately do not change management are ordered. The readmission rate to a hospital in the immediate postoperative was reported to 0.87% and compared favorably to the readmission rates reported in traditional microdiscectomy (4.1% to 5.8%) [20]. Yeung et al. reported the incidence of postoperative dysesthesia at 9.7% in his series of 176 patients [10,12]. Nellensteijn et al. corroborated these findings in his systematic review carried out in 2010 and concluded that comorbidities might impact the dysesthesia rate [54].

Hence, attempts to minimize the dysesthesia rate following an expertly executed transforaminal endoscopic decompression are highly relevant to the patients’ perception of the endoscopic procedure. Patient satisfaction may decrease when a bothersome dysesthesia sets in after an initial 5 to 10 days postoperative interval of pain relief. The authors investigated the feasibility of employing intraoperative neuromonitoring during the initial endoscopic working cannula position to avoid incidental compression of the exiting nerve root at the surgical level. That part of the procedure is typically not directly visualized. The authors found that neuromonitoring may detect such compression of the exiting nerve root and resolve it with the reposition of the working cannula during the initial steps of the endoscopic procedure. The detected SSEP and EMG neuromonitoring events with decrease amplitude and latency resolved with withdrawing and repositioning the working cannula and by waiting until these SSEPs and EMG signals had returned to baseline waves. Typically, such waiting times were on the order of one to two minutes but on average 1.15 min. Occasionally, the surgeon had to pause for up to three minutes before being able to commence the percutaneous endoscopic decompression procedure. Only in those patients where the SSEP and EMG signals did not return to baseline, the surgeons performed a foraminoplasty involving resection of the tip of the superior articular process with the intent of diminishing the presumed nerve root compression associated with advancing the endoscopic working cannula into Kambin’s triangle—the safe zone formed by the exiting and traversing nerve root and the pedicle of the inferior vertebral body. This has been corroborated by Jun-Song-Yang et al. who stated that foraminoplasty could be essential and necessary to facilitate the approach to a foraminal hernia, avoiding the manipulation and compression of the dorsal root ganglion and the symptomatic compressed nerve roots. The latter are typically inflamed, irritable, and susceptible to additional injury. Therefore, using the information obtained from the intraoperative neuromonitoring has the potential to decrease the incidence of neurological dysfunction associated with the transforaminal approach [55]. In this study, the intraoperative execution of this protocol has notably reduced the dysesthesia rate by one third in comparison to the rate observed in the control group, where the authors performed the same surgery without intraoperative monitoring.

While the application of neuromonitoring to improve outcomes with the outpatient transforaminal endoscopic decompression procedure is uncommon to current routine clinical practice with surgeries being done under MAC local anesthesia, it is frequently employed in traditional open and other forms of minimally invasive spinal surgery in the United States and the world over. From 2008 to 2014, the number of neuromonitored spine cases increased from 31,762 to 125,835, signifying a 296% increase over one decade [56]. Its benefit in reducing neurological morbidity has been demonstrated during spinal cord decompression and deformity surgery [30,33,34,56]. The benefits far outweigh the risk of routine neuromonitoring since complications with it are extremely rare [57]. Nonetheless, the added cost has been recognized [58,59], questioning its routine use during low-risk lumbar decompressions [58]. However, the authors stipulated that its use would lower surgical risk and improve clinical outcomes with the transforaminal endoscopic discectomy when the procedure is performed under general anesthesia. This hypothesis seems to be corroborated by the findings of this study. While the authors have no direct explanation as to why the observed dysesthesia rate was lower with concurrent application of neuromonitoring in the experimental versus the control group, the most plausible one is that the ability to assess in real-time intraoperatively whether their surgical access creates additional compression and to then act on that information by changing the course of the transforaminal endoscopic operation is likely the most relevant reason. As a result, the risk of injury to the exiting nerve root on top of what may have been caused by the herniated disc already may be lower. Not knowing the exact mechanism(s) of provoking postoperative dysesthesia is also one of the most significant limitations of the authors’ study besides a small sample size in both the experimental neuromonitoring and the control groups. The authors had no way to determine whether the risk for postoperative dysesthesia was solely determined by the surgical compression of the exiting nerve root, or whether there were any confounding factors, such as the size and location of the disc herniation, the duration of symptoms, extent of root ischemia, and duration of the neural element compression. Additionally, comorbidities, such as neuropathy, metabolic disease, including diabetes or renal disease, or age may also have played a role. Unfortunately, the statistical power of this feasibility study was insufficient to determine whether postoperative dysesthesia is more frequent in older than in younger patients. However, such an association appears likely since older patient suffer from more advanced degenerative changes of the facet joint complex, which may contribute to foraminal stenosis. More rigorous manipulation of the exiting nerve during stenosis decompression may increase the dysesthesia rate.

The implication is that the use of intraoperative neuromonitoring when applied during transforaminal endoscopic decompressions under general anesthesia may be appropriate and can lower the dysesthesia rates with the endoscopic decompression procedure. Future studies should go beyond the scope of this feasibility study in regards to the number of patient as we were unable to demonstrate a statistically significant benefit. Moreover, a more detailed subgroup analysis should be performed to validate further the conclusion of this retrospective study carried on the cases of five surgeons from four countries. Surgeon training and skill level may certainly also impact the dysesthesia rate, and again the authors had no reasonable way to quantify that to facilitate extrapolation of their experience to other clinical sites.

## 5. Conclusions

Neuromonitoring enabled the intraoperative detection of root compression of the exiting nerve root by the endoscope access cannula and by surgical manipulation. Its application resulted in a three-fold reduction of postoperative dysesthesia in patients undergoing videoendoscopic transforaminal discectomy under general anesthesia. The combined 65-year clinical experience of the three senior authors shows that neuromonitoring is not mandatory, particularly when the transforaminal endoscopic is done under local anesthesia with direct patient feedback, but may be considered by those surgeons who perform it under general anesthesia.

## Figures and Tables

**Table 1:** Surgical level distribution in patients who underwent transforaminal endoscopy (*n* = 65).

Level	Number of Patients	Percent	Cumulative Percent
L2-L3	1	1.5	1.5
L3-L4	6	9.2	10.8
L3-L5	4	6.2	16.9
L4-L5	29	44.6	61.5
L4-S1	10	15.4	76.9
L5-S1	15	23.1	100.0
Total	65	100.0	

**Table 2:** Results of paired T-testing comparing means of preop to postop Oswestry disability index (ODI), visual analog scale (VAS)-leg and back.

	Mean	Standard Deviation	Standard Error Mean	95% Confidence Interval		t	Degree of Freedom	Significance (2-Tailed)
				Lower	Upper			
ODI-Preop–ODI-Postop	12.692	13.122	1.628	9.441	15.944	7.798	64	<0.0001
VAS-Back Preop–VAS-Back Postop	1.723	1.206	0.150	1.424	2.022	11.523	64	<0.0001
VAS-Leg Preop–VAS-Leg Postop	7.708	1.902	0.236	7.236	8.179	32.677	64	<0.0001

**Table 3:** Results of paired T-testing comparing intra- and postoperative mean voltage changes as a result of the transforaminal endoscopic decompression.

Neuromonitoring Modality	Mean Voltage (MV)		Number of Patients	Standard Deviation		Standard Error Mean		
SSEP Intraoperatively	11.82		33	1.845		0.321		
SSEP Postoperativly	15.36		33	1.245		0.217		
TCMEP Intraoperatively	10.61		33	1.413		0.246		
TCMEP Postoperatively	17.64		33	0.962		0.168		
	Mean Voltage	Standard Deviation	Standard Error Mean	95% Confidence Interval		t	df	Significance (2-tailed)
				Lower	Upper			
SSEP MV Intra–SSEP MV Postoperatively	−3.545	2.223	0.387	−4.334	−2.757	−9.161	32	<0.0001
TCMEP MV intra–TCMEP MV Postoperatively	−7.030	1.311	0.228	−7.495	−6.566	−30.814	32	<0.0001

SSEP—somatosensory evoked potentials; TCMEP—transcranial motor evoked potentials; MV—mean voltage; df—degree of freedom.
